# Simplification to co-formulated rilpivirine/emtricitabine/tenofovir in virologically suppressed patients: Data from a multicenter cohort

**DOI:** 10.7448/IAS.17.4.19812

**Published:** 2014-11-02

**Authors:** Carmela Pinnetti, Simona Di Giambenedetto, Franco Maggiolo, Patrizia Lorenzini, Massimiliano Fabbiani, Chiara Tommasi, Alessandra Latini, Adriana Ammassari, Laura Loiacono, Gaetana Sterrantino, Rita Bellagamba, Evangelo Boumis, Andrea Antinori, Mauro Zaccarelli

**Affiliations:** 1 Clinical Department, National Institute for the Infectious Diseases, Rome, Italy; 2 Department of Infectious Diseases, Catholic University of the Sacred Heart, Rome, Italy; 3 Division of Infectious Diseases, Ospedali Riuniti, Bergamo, Italy; 4 Department of Dermatology and Infectious Diseases, San Gallicano Institute, Rome, Italy; 5 Division of Infectious Diseases, ‘Careggi’ Hospital, Florence, Italy

## Abstract

**Background:**

To assess efficacy and safety of treatment simplification to co-formulated Rilpivirine/Emtricitabine/Tenofovir (RPV/FTC/TDF) in virologically suppressed patients.

**Materials and Methods:**

Endpoints of the analysis were: (a) treatment discontinuation of RPV/FTC/TDF for any reasons and (b) occurrence of virological failure (VF) defined as confirmed HIV-RNA >50 cp/mL).

**Results:**

Overall, 508 patients from five Italian reference centres were included: male gender 71.9%; median age 47 years (IQR 40–52); IVDU as HIV risk 17.7%; HCV-AB positive 23.4%; CDC-C stage 17.5%; median CD4 cells/µL at switch 655 (IQR: 487–843); median number of previous regimens three (IQR 2–7). In a median follow-up of 196 days (IQR: 84–287), 31 patients discontinued RPV/FTC/TDF (virological failure n=5, hypersensitivity reaction n=2, GI-toxicity n=6, liver toxicity n=1, CNS-toxicity n=4, kidney toxicity n=5, patient's decision/lost in follow-up n=10). Moreover, VF occurred in eight patients (five discontinued regimen, while three remained on RPV/FTC/TDF). At survival analysis, the probabilities of treatment discontinuation or VF were 5.5% and 1.2% at 6 months, 13.2% and 2.8% at 12 months, respectively (Figure 1). At adjusted Cox model, factors associated with discontinuation were: <200 CD4 cells/µL at switch (HR 5.3, 95% CI 1.1–25.9, p=0.038), number of pre-switch regimens (for each extra regimen: HR 1.05, 95% CI 1.01–1.10, p=0.024), male gender (HR 0.4, 95% CI 0.2–0.9, p=0.032). Only the number of pre-switch regimens was associated with VF (HR 1.13, 95% CI 1.06–1.21, p=0.001). Type of pre-switch regimen was not associated with discontinuation or failure, but no VF was observed if switching from co-formulated Efavirenz/FTC/TDF or from Raltegravir containing regimens. Switching to RPV/FTC/TDF led to significant improvement in fasting lipids levels: the decrease in cholesterol, LDL and triglycerides was observed switching from any regimen, but was more marked from boosted PI. In contrast, a moderate increase in transaminase (switching from all regimens except NNRTI-containing) and creatinine (except from TDF-containing regimens) were observed.

**Conclusions:**

Our data suggest that switching to RPV/FTC/TDF in virologically suppressed patients could be a good strategy with low risk of virological failure or treatment discontinuation; the switch is also associated with significant improvement in lipid profile.

**Figure 1:**
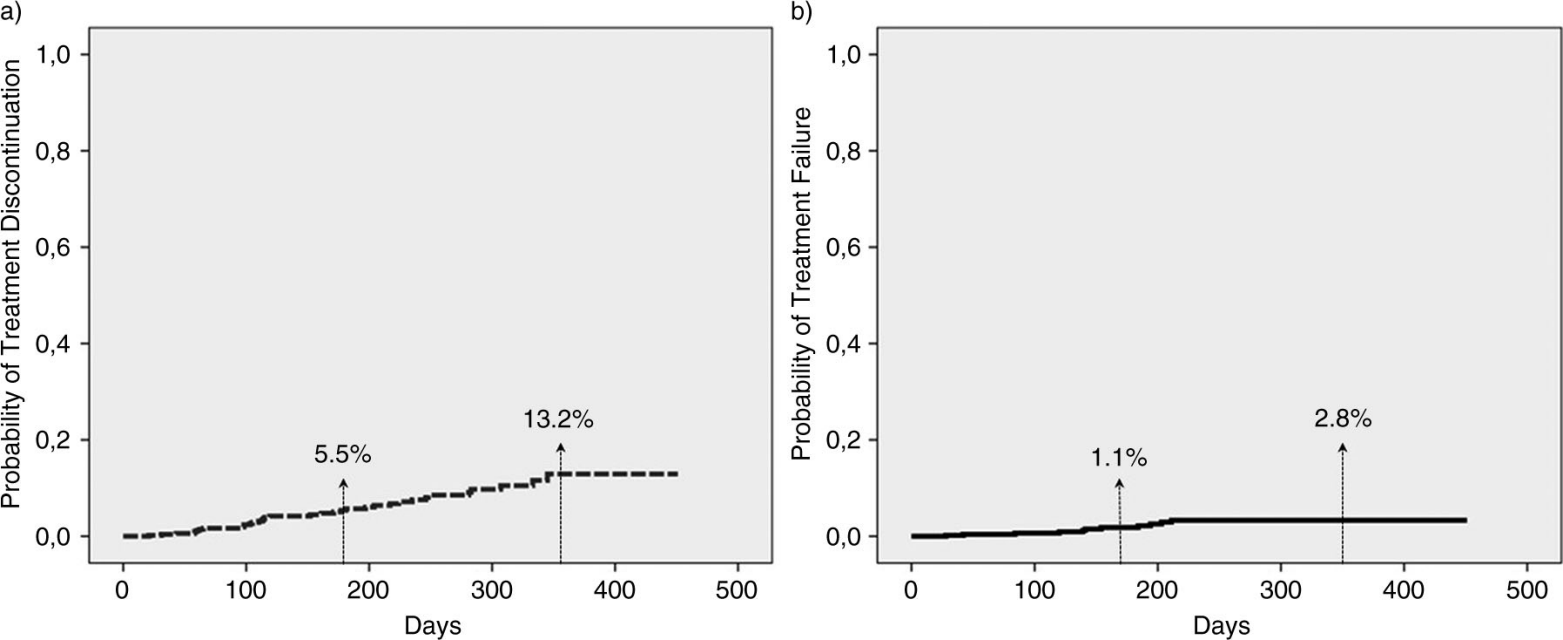
Probability of a) treatment discontinuation; b) virological failure at 6 and 12 months of patients treated with co-formulated rilpivirine/emtricitabine/tenofovir for treatment simplification.

